# The Proline Rich Homeodomain Protein PRH/Hhex Forms Stable Oligomers That Are Highly Resistant to Denaturation

**DOI:** 10.1371/journal.pone.0035984

**Published:** 2012-04-23

**Authors:** Anshuman Shukla, Nicholas M. Burton, Padma-Sheela Jayaraman, Kevin Gaston

**Affiliations:** 1 Institute for Biomedical Research, Birmingham University Medical School, Edgbaston, Birmingham, United Kingdom; 2 School of Biochemistry, University of Bristol, Bristol, United Kingdom; Université Paris-Diderot, France

## Abstract

**Background:**

Many transcription factors control gene expression by binding to specific DNA sequences at or near the genes that they regulate. However, some transcription factors play more global roles in the control of gene expression by altering the architecture of sections of chromatin or even the whole genome. The ability to form oligomeric protein assemblies allows many of these proteins to manipulate extensive segments of DNA or chromatin via the formation of structures such as DNA loops or protein-DNA fibres.

**Principal Findings:**

Here we show that the proline rich homeodomain protein PRH/Hhex forms predominantly octameric and/or hexadecameric species in solution as well as larger assemblies. We show that these assemblies are highly stable resisting denaturation by temperature and chemical denaturants.

**Conclusion:**

These data indicate that PRH is functionally and structurally related to the Lrp/AsnC family of proteins, a group of proteins that are known to act globally to control gene expression in bacteria and archaea.

## Introduction

The nucleus is highly compartmentalised despite the absence of membrane bound organelles. The dynamic self-organisation of sub-nuclear domains or nuclear bodies is essential for the establishment and maintenance of nuclear architecture and ultimately for gene regulation (reviewed by Misteli [1]). Architectural nuclear proteins include the lamins and scaffolding factors such as SatB1 and PML [2], [3]. Some of these proteins bind to DNA directly, as in the case of SatB1 [3], while others are recruited to DNA via partner proteins or appear to act independently of DNA. Many of these architectural proteins self-associate and their recruitment to chromatin can result in the *de novo* formation of macromolecular structures such as Cajal bodies and PML bodies with the further and simultaneous recruitment of additional components including RNAs [4], [5]. However, the distinction between architectural proteins and transcription factors is not clear cut since some proteins that are generally considered to be transcription factors also function in this manner; forming self-associating complexes that compact or otherwise manipulate chromatin [6]. In this way architectural proteins and transcription factors can control the chromatin loop landscape to regulate the expression of individual genes or groups of genes. The mis-regulation of many of these proteins leads to alterations in gene expression and to diverse disease states [2].

**Figure 1:**
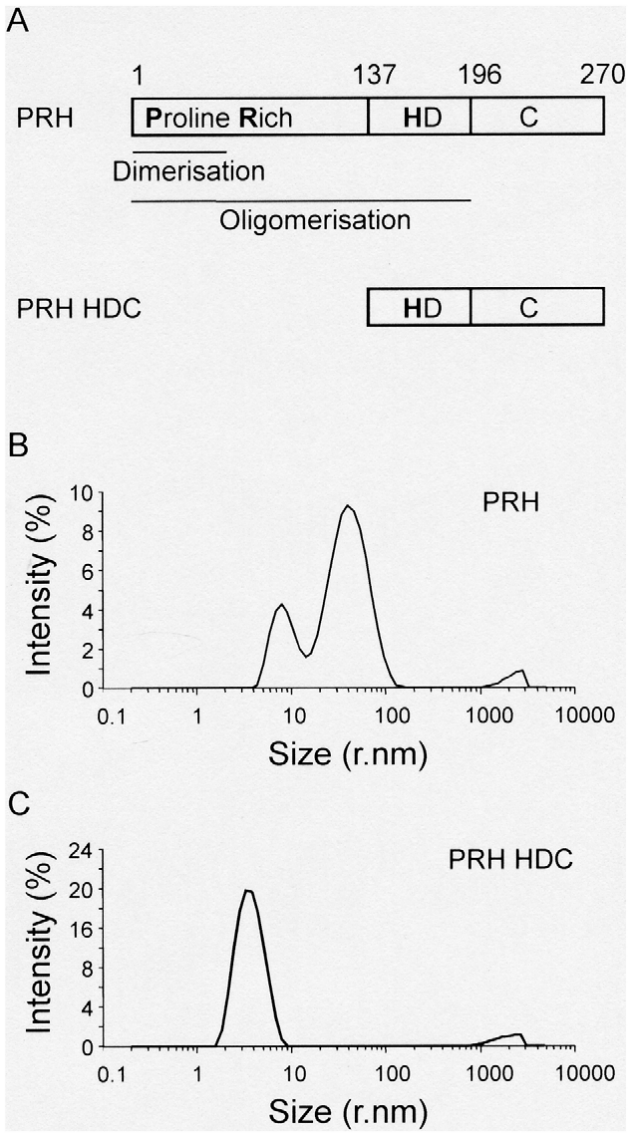
PRH oligomers predominate in solution. (A) A schematic representation of the PRH protein and its functional domains. Proline Rich indicates the transcriptional repression domain, HD indicates the homeodomain and C indicates the C-terminal domain. The PRH HDC protein lacks the N-terminal domain. The intensity size distribution of PRH particles (B) and PRH HDC particles (C) in solution determined using DLS.

The Proline-Rich Homeodomain protein (PRH, also known as Hhex) is an essential DNA binding protein that plays multiple roles in embryonic development and in the adult (reviewed by Soufi and Jayaraman [7]). In the developing embryo PRH regulates body axis formation and the formation of several organs including the liver, pancreas, heart and thyroid, the vasculature, and the haematopoietic system. In the adult, PRH regulates multiple steps in haematopoiesis and controls cell growth. Mutations that result in mis-expression, or mis-localisation of PRH are associated with leukaemias as well as thyroid and breast cancers [8]–[11]. PRH can activate or repress the transcription of its target genes and it can also regulate gene expression post-transcriptionally by interacting with translation factor eIF4E [12]–[14]. Crosslinking studies have shown that PRH forms homo-oligomeric complexes in cells and *in vitro* [15]. The PRH protein is around 270 amino acids in length and consists of three regions: a proline-rich N-terminal transcription repression domain required for oligomerisation and binding to eIF4E and other PRH-partner proteins, a central DNA binding homeodomain that mediates binding to DNA and an acidic C-terminal domain that mediates transcription activation (Fig. 1A). The first 46 amino acids of PRH form a novel dimerisation motif while the region between amino acids 46 and 132 is capable of interacting with the PRH homeodomain and is probably important during oligomerisation when it is thought to mediate intermolecular contacts [15]. PRH oligomers bind to arrays of suitably spaced PRH sites on DNA and the protein does not appear to form monomers or any other multimers smaller than an octamer [16]. This suggests that PRH binds to multiple sites within its target promoters *in vivo* and in support of this idea we have shown that several PRH-regulated genes including the Goosecoid gene and the Vegfr-1 and Vegfr-2 receptor genes, contain clustered arrays of sites that mediate PRH binding [17]. We do not yet know whether the protein forms DNA loops or whether the protein assembly can wrap DNA around itself. However, our KMnO_4_ footprinting data has shown that PRH induces pronounced DNA distortions on binding to a target promoter [16].

Gel filtration chromatography and analytical ultracentrifugation (AUC) have failed to detect monomeric PRH species suggesting that PRH oligomers are the native form of this protein [15]. AUC demonstrated that PRH forms oligomers of different sizes; discrete disc shaped octameric complexes and more spherical double octamers, as well as larger multimers [6], [15]. PRH double octamers bind to DNA in an ordered fashion and linear dichroism (LD) and electron microscopy (EM) have shown that when these oligomers bind to PRH sites they significantly compact the DNA and form protein arrays or fibres [6]. This suggests that PRH is similar to members of the Lrp/AsnC family of proteins found in bacteria and archaea, proteins that are known to form octameric and hexadecameric species but which are thought to have no equivalents in eukaryotes [18], [19]. Here we show that in solution PRH predominantly forms particles that are octamers or hexadecamers and that these particles are highly resistant to thermal and chemical denaturation. These data suggest that PRH forms obligate oligomers *in vivo*.

## Results

### PRH exists as octamers and larger polymers in solution

We have shown previously using analytical ultracentrifugation (AUC) that PRH comprises species with sedimentation coefficients of 9S and 25S as well as multiple species with sedimentation coefficients larger than 40S [6]. Although the purified full length protein carries a histidine tag, this sequence is not responsible for oligomerisation since crosslinking studies have shown that PRH proteins lacking this sequence form oligomers *in vivo* [15]. The 9S species is an oblate spheroid that most likely corresponds to a PRH octamer whereas the 25S species is a more spherical particle that most likely corresponds to a double octamer. The multiple species larger than 40S have molecular weights in excess of 1 MDa which suggests that they are more highly polymerised forms of PRH. Electron microscopy reveals the existence of bead-like particles with radii of around 15–20 nm that could be octamers or double octamers and larger polymeric extended structures [6]. However, these studies cannot report on the relative amounts of each of these species in solution. To address this question and to further characterise these species we have examined the PRH protein in solution using dynamic light scattering (DLS). The intensity size distribution of particles obtained in DLS is a plot of the relative intensity of light scattered by particles of various sizes. Intensity is proportional to (particle size)^6^ so equal numbers of particles that differ in size by a factor of 10 will result in two peaks that differ in intensity by 10^6^. The hydrodynamic radius that is calculated in DLS refers to the diameter of a sphere that has the same translational diffusion coefficient as the particle.

We purified His-tagged full length PRH and examined this protein using DLS. The protein produces an intensity size distribution with two major peaks (Fig. 1B). This indicates that PRH exists predominantly as two discrete populations, one with a hydrodynamic radius distribution of around 8.6 nm and one with a hydrodynamic radius distribution of around 43 nm. Both of these peaks are broad indicating that they could be made up of multiple species. In addition there is a relatively small population with around 500 nm radius distribution. Since intensity is proportional to the diameter^6^ we conclude that the predominant species in the sample is the 8.6 nm particle. Although the 8.6 nm particle is the major component, it is not possible to quantify the relative amounts of the different populations from this data. Assuming that the particles are globular, the predicted molecular weight of the 8.6 nm radius particle is 517 kDa and this is close to the calculated molecular weight of a PRH double octamer (582 kDa). AUC suggests that PRH octamers and double octamers have the same diameter and differ only in that the latter are spherical whereas the former are oblate. Therefore, these species would have the same hydrodynamic radius. The 43 nm particles have a predicted molecular weight of around 22,300 kDa suggesting that they are highly oligomeric. However, rigid body association of two 8.6 nm radius spheres produces an apparent hydrodynamic radius of 17.2 and an apparent molecular weight of 2,618 kDa and the association of three, four or five spheres produces apparent molecular weights of 6,762 kDa, 13,256 kDa and 22,344 kDa, respectively. Species of this nature would be entirely consistent with previous data obtained using AUC and electron microscopy [6]. The largest species (∼500 nm) is not highly populated and it is not removed by centrifugation or filtration; it is therefore not a non-specific aggregate (data not shown). Most likely it corresponds to highly oligomeric protein assemblies. The large size of the oligomeric species, their heterogeneity, and the low occupancy of the largest states, have made it difficult to examine these species using AUC and we are unable to provide equilibrium constants for these associations.

In marked contrast to the full length protein, a truncated PRH protein which lacks the N-terminal proline rich domain (PRH HDC) produces an intensity size distribution with a single broad peak corresponding to a hydrodynamic radius distribution of around 3.3 nm (Fig. 1C). This confirms that the N-terminal domain is required for the formation of large PRH oligomers. The 3.3 nm particles have a predicted molecular weight of around 53 kDa suggesting that the truncated protein could be dimeric. However, previous AUC experiments have shown that the PRH HDC is a monomer in solution [15] indicating that the protein could be an elongated monomer. We conclude that the full length PRH protein is polydisperse and that the predominant species in solution is most likely the PRH octamer or double octamer although larger oligomers are certainly present in smaller amounts.

### PRH has well defined secondary structural composition

**Figure 2:**
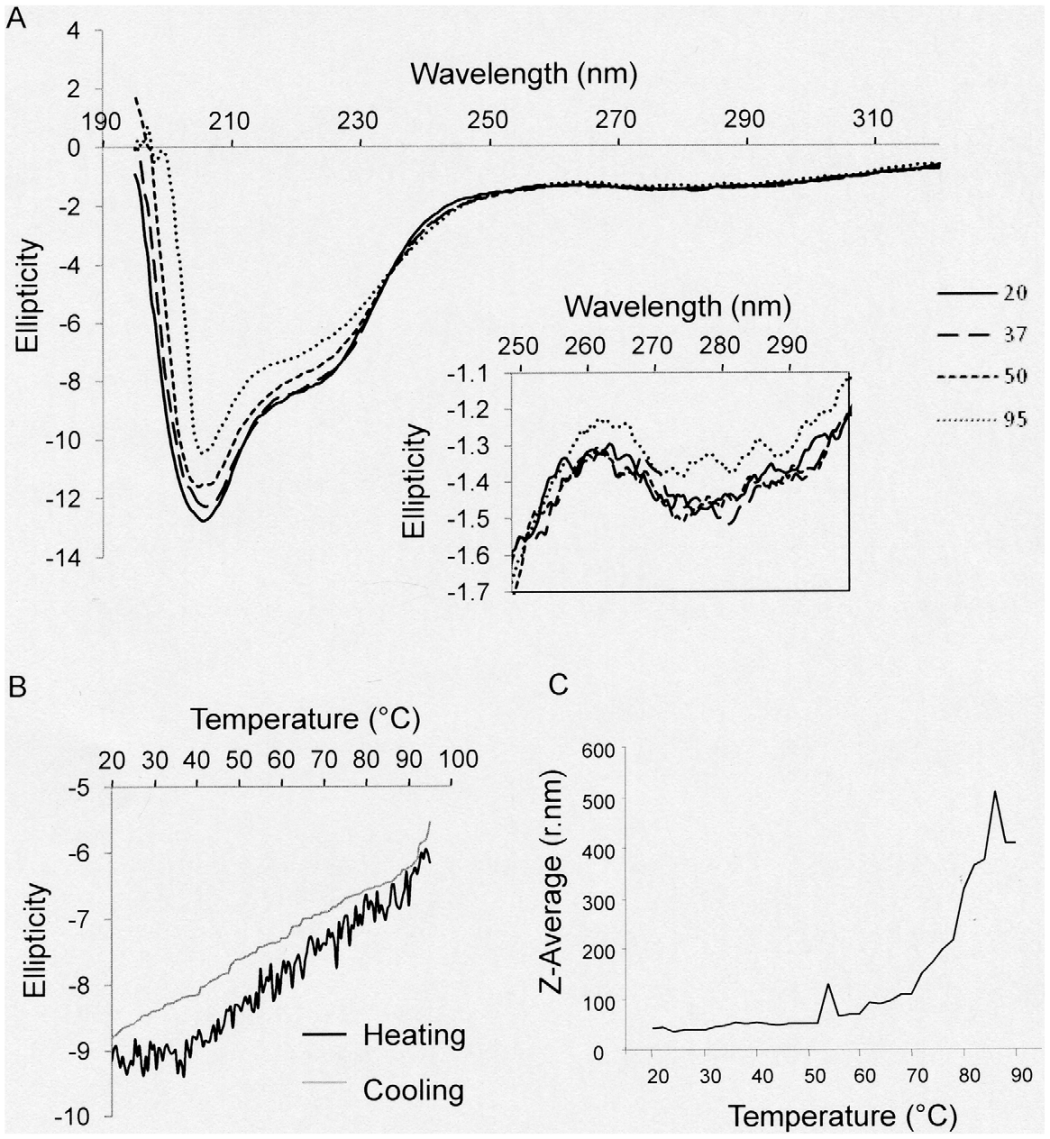
PRH oligomers are thermostable. (A) Circular dichroism was used to examine the secondary structure of the full length PRH protein at the temperatures shown. The insert shows the near-UV circular dichroism spectra at the temperatures shown. (B) The far-UV circular dichroism signal at 200 nm measured while increasing (black line) and then decreasing (grey line) the temperature. (C) The mean diameter of PRH particles at increasing temperature determined using DLS.

We have previously reported the far-UV circular dichroism (CD) spectra of the isolated PRH homeodomain and C-terminal region and the isolated PRH N-terminal domain. The PRH N-terminal domain appears to be an extended flexible structure with significant random coil and/or intrinsic disorder whereas the homeodomain and C-terminal region appears to be largely α helical. However, the full-length protein has not been characterized using CD and it is possible that the N-terminal random coil region might become folded in the oligomeric state. The far-UV CD spectra of the full length PRH protein in PBS at 20°C indicates significant α helical content as characterised by minima at 208 nm and 222 nm respectively (Fig. 2A). There is no evidence of significant β sheet in the full length protein since there is no characteristic peak at 215 nm and the negative ellipticity observed at 215 nm is very likely due to the overlapping of minima at 222 nm and 208 nm Based on these observations we can conclude that the protein has α-helical content perhaps largely limited to the homeodomain and significant random coil and/or intrinsic disordered domain composition.

### PRH is resistant to unfolding by heat

To examine to thermal denaturation of PRH we obtained far and near-UV CD spectra at temperatures between 20°C and 95°C. There was no change in the protein secondary structure at temperatures below 50°C. At 50°C there are minor changes to the far-UV CD spectrum around 195 nm but little or no change at 208 and 222 nm (Fig. 2A). This suggests that although there may be some alterations in the disordered regions of the protein (possibly due to increased ordering), there is little alteration to the α-helical regions. At 95°C a similar CD spectrum is observed although there are significant decreases in negative ellipticity at 208 nm and 222 nm. This suggests that the homeodomain stays folded and undergoes only minor changes at higher temperatures while the N-terminal unstructured regions, which are responsible for a near zero ellipticity around 195 nm, undergo structural reorganization. Near-UV CD spectra confirm that there is no unfolding of the hydrophobic core of the protein at 95°C (Fig. 2A inset panel). The structural changes that are seen at high temperatures are reversible since the reduction in negative ellipticity is reversible on cooling; the far-UV CD signal at 200 nm declines with increasing temperature although negative ellipticity is retained at 95°C (Fig. 2B) and the lost negative ellipticity is regained on cooling. DLS at increasing temperatures shows that the average particle size increases above 60°C suggesting that there is increased oligomerisation (Fig. 2C). However, samples heated to 95°C and then cooled to 20°C do not show increased amounts of the highly oligomeric species in DLS confirming that the effects of heating are reversible (data not shown). We conclude that PRH secondary structure is not significantly perturbed by heating and cooling and that the structural reorganisations short of unfolding that are seen at high temperature are reversible. Although it is possible that the His-tag has an effect on the stability of the protein, this is unlikely since the tag does not mediate oligomerisation.

**Figure 3:**
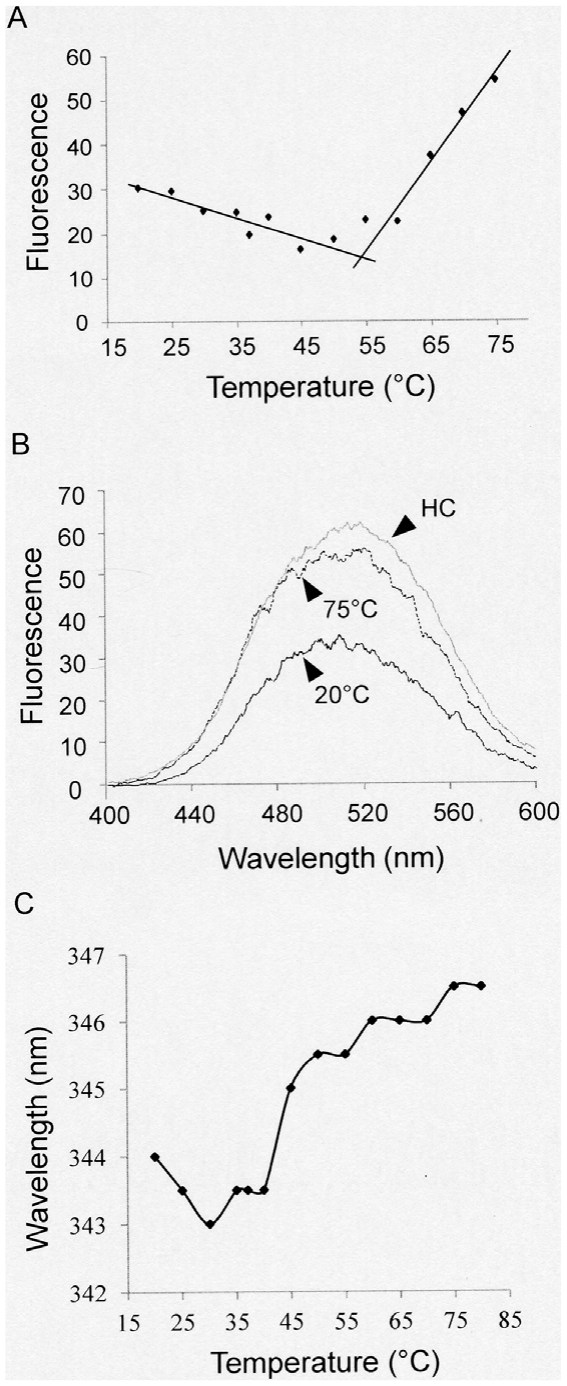
Changes in PRH structure at high temperature. (A) PRH was incubated with ANS before emission fluorescence spectra were collected between 20°C and 75°C. (B) PRH was incubated with ANS and emission fluorescence spectra were collected between 400 nm and 600 nm at 20°C and 75°C and after heating to 75°C and then cooling to 20°C (HC). (C) Fluorescence emission spectroscopy was performed to monitor intrinsic tryptophan fluorescence between 20°C and 75°C.

To examine the structural changes in PRH at high temperatures in more detail we made use of the fluorescent hydrophobic dye 8-anilino-1-naphthalene sulfonate (ANS). This dye is used widely as a probe for the presence of clusters of hydrophobic residues on the surfaces of proteins and to examine the formation of partially structured folding/unfolding intermediates. Fluorescence spectra showed ANS fluorescence between 20°C and around 60°C and above 60°C increased ANS fluorescence is observed suggesting increased ANS binding (Fig. 3A). This is unlikely to be due to protein unfolding since there is little change in the CD spectra at these temperatures. It is also unlikely to result from dissociation of the quaternary structure of the protein into smaller particles since DLS shows increased oligomerisation at these temperatures. Interestingly, the heated and then cooled protein (HC) retains increased ANS binding (Fig. 3B). Taken together these data suggest that at high temperatures small conformational changes and possibly increased oligomerisation result in increased ANS binding. They also suggest that bound ANS is not released on cooling. It is also important to note that ANS binds to PRH even at 20°C. Therefore, the hydrophobics in the protein are relatively exposed to ANS even before any structural changes occur during heating.

PRH contains three tryptophan residues. W126 is located in the N-terminal region of PRH, whereas W184 and W192 are located within the DNA recognition helix of the homeodomain and at the end of the recognition helix, respectively. Our previous studies suggested that W126 may be in close proximity to W184 and W192 as the C-terminal region of the PRH repression domain (1–141) is known to interact with the homeodomain (141–200) [15]. Tryptophan fluorescence emission wavelength is sensitive to the polarity of the tryptophan micro-environment. Increased exposure of the tryptophans in PRH to solvent would be expected to result in a red shift of the emitted wavelength. Although the wavelength of maximum emission is shifted to the red at 45°C and higher temperatures, the change is gradual (Fig. 3C). Moreover, it does not reach a plateau that would indicate full exposure to solvent. This small change therefore suggests that one or more of the tryptophans are more exposed at high temperature. These data confirm that PRH undergoes structural rearrangements but not unfolding at high temperature and that these changes are reversible on cooling.

### PRH is resistant to chemical denaturation

**Figure 4:**
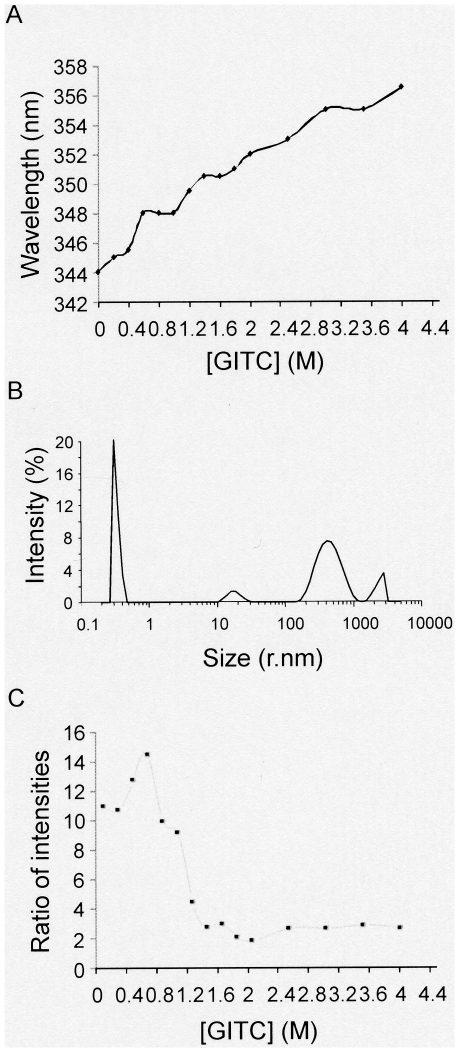
Changes in PRH structure in the presence of chemical denaturant. (A) Fluorescence emission spectroscopy was performed in the presence of increasing concentrations of GITC. (B) The intensity size distribution of PRH particles in the presence of 4 M GITC determined using DLS. (C) The ratio of the maximum intensities of the major PRH peaks obtained using DLS in the presence of increasing concentrations of GITC.

The native PRH protein shows maximum tryptophan emission at 344 nm (Fig. 4A). We used the chaotropic agent guanidinium thiocyanate (GITC) to examine changes in PRH in the presence of increasing concentrations of this chemical denaturant. Incubation with low concentrations of GITC produced little alteration in the tryptophan emission spectra but at 0.5 M GITC there is a shift to an emission maximum at 348 nm. There is little change between 0.5 M and 1.2 M GITC and then there is a progressive shift to 356 nm at 4 M GITC (Fig. 4A). These data indicate that the tryptophans are protected from exposure to solvent until 0.5 M GITC and thereafter there are further gradual alterations in the tryptophan microenvironment. One possible explanation for these observations is that the lower concentrations of GITC bring about the rearrangements of PRH subunits or domains within the oligomers similar to those brought about by high temperature. Although GITC concentrations above 2 M might be expected to bring about the dissociation of PRH particles to smaller units such as tetramers or dimers, the fact that tryptohan emission does not plateau again suggests that PRH still retains some structure even at 4 M GITC. This non-cooperative transition could also indicate that the structure is not cooperative or more likely that the population is not homogeneous.

To examine the oligomersiation of PRH in the presence of GITC we examined light scattering at the same GITC concentrations used for fluorescence studies. There is a modest increase in the size distribution of smaller PRH particles in GITC and this may be accounted for by the change in the ionic strength of the solution or by localised protein unfolding (Fig. 4B). In contrast, the less abundant larger particles appear to significantly increase their size distribution at higher GITC concentrations. This suggests that GITC could cause some polymerisation or aggregation of the larger PRH particles. Evidence to support this conclusion comes from the ratio of the intensity of the peaks for the smaller and larger particles. At GITC around 1 M there is a sharp transition due to the accumulation of the larger species (Fig. 4C). It should be noted that breakdown of PRH particles to monomers would not be detected easily by DLS as smaller particles produce very small peak intensity size distributions.

## Discussion

Previous studies have shown that PRH forms protein oligomers *in vitro* and *in vivo*. These oligomers appear to be octameric complexes, double octamers and larger multimers [6], [15]. However, these studies have not reported on the relative amounts of each species. Here we have shown that PRH forms predominantly octameric and/or hexadecameric species in solution. This suggests that these species are a biologically relevant form of PRH. The formation of these oligomers is certainly consistent with the proposed binding of this protein to linear arrays of PRH binding sites near PRH target genes [6].

Oligomeric structural proteins such as collagen that play mechanical roles can be highly resistant to denaturation by temperature or chemical agents. This is unsurprising given that these proteins are involved in diverse processes that require a high degree of tensile strength. Interactions between the subunits in these oligomers play a large part in the structural stability of these assemblies. Oligomeric non-structural proteins can also be resistant to denaturation. Chaperonin 10 heptamers and p53 tetramers, for example, are resistant to thermal denaturation and in these cases resistance to unfolding also arises in part at least as a consequence of oligomerisation [20], [21]. Here we have shown that PRH oligomers are highly resistant to unfolding by heat and chemical denaturants. However, the stability of PRH does not arise simply from its oligomeric state. Many oligomeric transcription factors form highly dynamic assemblies that are easily disassembled. For example, under physiological conditions histone proteins form stable octameric nucleosomes only in the presence of DNA [22]. Rather, the resistance to denaturation shown by PRH suggests that this protein forms obligate oligomers in which there are large hydrophobic interacting surfaces, and possibly the exchange of secondary structure units or domain swaps, between monomers. This produces oligomers in which dissociation of the subunits and protein unfolding are coupled and it could explain why we have not observed PRH assemblies smaller than octamers in AUC or gel filtration chromatography [6], [15]. In contrast, in more dynamic assemblies, the subunits usually interact and fold independently with the result that oligomer dissociation does not required unfolding of the monomers and there can be rapid subunit exchange. The stability of the PRH oligomers therefore suggests that they do not undergo this type of subunit exchange but instead form stable oligomers *in vivo*. These multivalent platforms could allow the recruitment of multiple PRH-partner proteins to PRH target genes in order to regulate transcription in response to multiple signals.

The structural basis for the high thermostability of some proteins is not well understood. However, studies of conserved proteins that are found in both thermophiles and mesophiles suggest that a small number of addition interactions between the subunits in a protein can greatly increase thermal stability [23]. Consistent with this view a single amino acid substitution that facilitates hydrophobic packing at the dimerisation interface in the LacR protein dramatically increases thermostability [24]. It would seem likely that in the case of PRH thermostability arises as a result of multiple interactions between the subunits as well as from the inherent properties of the component structural domains of this protein. Despite their structural stability PRH proteins can undergo a substantial degree of protein reorganization. Although there is no unfolding of the hydrophobic core of the protein at elevated temperatures, structural rearrangements do occur, most likely at interfaces between the interacting subunits or involving the local denaturation of individual subunits. Similarly, at low concentrations the chemical denaturant GITC brings about subtle rearrangements of PRH subunits or domains within the oligomers. These data suggest that despite the high stability of the PRH oligomer it is not a static platform for the assembly of a protein-DNA complex. It would seem likely that each subunit within the PRH oligomer undergoes independent structural changes that could include some local unfolding. This would result in a dynamic ensemble of PRH states in which subunits could be in different conformations that include locally unfolded regions. Protein modifications such as phosphorylation [25] and the binding of PRH-interacting proteins such as the TLE corepressor proteins [13], would be expected to induce conformational changes within the PRH oligomer analogous to the changes seen at high temperature and these changes could alter both DNA binding and transcriptional regulation.

In terms of its overall properties PRH is strikingly similar to members of the Lrp/AsnC family of transcription factors found in bacteria and archaea. These proteins also form octameric and hexadecameric assemblies that bind to arrays of suitably spaced sites on DNA in order to control gene expression and/or genome architecture. The Lrp/AsnC oligomers are thought to form disc-like proteins with dimeric helix-turn-helix domains arranged around the perimeter [18], [19]. PRH octamers also appear to form disc-like structures and since PRH is also a DNA binding protein, we can assume that the PRH homeodomains are also localized to the surface of the oligomer and possibly on the perimeter of the disc. Outside of the homeodomain, which is similar to the helix-turn-helix motif, there is no sequence homology between PRH and members of the Lrp/AsnC family and these proteins are unlikely to be evolutionarily related. PRH is not found in all eukaryotes and together with the absence of sequence homology to the Lrp/AsnC family this suggests that PRH did not arise from an ancestral Lrp-like protein found in the last common ancestor of bacteria, archaea and eukaryotes. However, PRH orthologues are found in chordates and hemi-chordates, suggesting that this protein is conserved over at least 550 million years. These data suggest that the ancestor of these deuterostomes acquired PRH when the PRH homeodomain was fused to a dimerisation domain that could also bind to the homeodomain and thereby allow the formation of octameric or hexadecameric complexes. This conferred upon PRH the ability to bind to arrays of homeodomain binding sites and the ability to bring about DNA compaction. The similarities between PRH and the Lrp/AsnC family therefore appear to represent a remarkable example of convergent evolution.

## Materials and Methods

### Protein purification

His-tagged full length human PRH protein and a truncated protein corresponding to the PRH homeodomain and C-terminal region (PRH HDC) were expressed in *Escherichia coli* BL21 cells and purified as described previously [15]. In brief, His-PRH and His-PRH-HDC were purified over Hi-trap nickel chelating columns (Amersham Pharmacia Biotech) using an AKTA FPLC system. The proteins were eluted using an imidazole gradient, snap frozen in 50 mM Tris (pH 8.5), 20% (v/v) glycerol containing protease inhibitor cocktail (Roche) and stored at −80°C until required.

### Dynamic Light Scattering

Measurements were done in a Malvern Instruments zetasizer nano in a quartz cuvette requiring 45 µl of sample. PRH was used at a concentration of 0.15 mg/mL (4.34 µM). The buffer used was phosphate buffered saline (PBS) with 20% glycerol and an additional 200 mM NaCl. For temperature measurements the cuvette was inserted into the unit and the temperature allowed to equilibrate for 2 minutes before a 300 s measurement was performed. For chemical denaturation studies samples were prepared individually and allowed to equilibrate for 30 minutes at 20°C after the addition of guanidinium thiocyanate (GITC). The samples were centrifuged for 10 minutes at 15,000 rpm in a microcentrifuge prior to analysis. For all measurements, the refractive index and viscosity of the sample were calculated (using the instrument specific Zetasizer 6.2 software, which takes into account the salt concentrations and other additives before calculating the refractive index and viscosity) and applied.

### Spectroscopy

Fluorescence spectra were collected on a PerkinElmer LS-50B spectrofluorimeter with variable excitation and emission bandpasses as appropriate using excitation with light of 285 nm and scanning protein emission between 300 and 450 nm. CD spectra were collected at intervals of 1 nm on a Jasco J-810 spectropolarimeter through scanning of wavelengths from 300 to 195 nm using a protein concentration of 0.08 mg/mL and a cuvette path length of 0.1 cm (requiring sample volumes of 200 µl). CD signals below 195 nm could not be collected because the spectra were noisy. For CD spectra at 20°C 20 accumulations were performed and averaged. For thermal studies the PRH was incubated at the required temperature for 5 minutes before a single spectrum was taken. In this case no accumulation scans were performed. The temperature was increased at the rate of 1°C/minute. ANS (8-anilino-1-naphthalene sulfonate) was used at a final concentration of 10ìM.
